# Biofilm and pathogenic factor analysis of *Gardnerella vaginalis* associated with bacterial vaginosis in Northeast China

**DOI:** 10.3389/fmicb.2022.1033040

**Published:** 2022-12-22

**Authors:** Xiaolu Ma, Xiaoxi Wang, Shengna Ye, Jinnan Liu, Hong Yuan, Nan Wang

**Affiliations:** ^1^Department of Clinical Laboratory Medicine, The First Affiliated Hospital of Dalian Medical University, Dalian, Liaoning, China; ^2^Department of Laboratory Medicine, Dalian Medical University, Dalian, Liaoning, China; ^3^Department of Clinical Laboratory Medicine, Dalian Municipal Central Hospital, Dalian, Liaoning, China

**Keywords:** *Gardnerella vaginalis*, biofilm, bacterial vaginosis, resistance, confocal laser scanning microscopy

## Abstract

**Introduction:**

*Gardnerella vaginalis* is a major pathogen responsible for bacterial vaginosis (BV). However, the recurrence of infection and the antibiotic resistance of biofilms remain significant challenges for the treatment of BV. In this study, we aimed to analyze the pathogenic factors and drug sensitivity associated with the clinical treatment of BV in Northeast China.

**Methods:**

Subgroups were identified by clade-specific polymerase chain reaction (PCR). Biofilm formation was measured by crystal violet staining, confocal laser scanning microscopy (CLSM) and scanning electron microscopy (SEM). The inhibition and eradication of biofilm formation were measured by XTT and broth recovery-based methods.

**Results:**

Of the 24 samples of *G. vaginalis*, 11 samples and American Type Culture Collection (ATCC) 14018 formed biofilms; the remainder did not. The positive rates of detection for the *sialidase A* and *vly* genes in the 24 *G. vaginalis* samples were 100% and 79.2%, respectively. Moreover, 21 samples (87.5%) showed resistance to metronidazole and 16 (66.7%) presented with sensitivity towards clindamycin. The biofilm MIC_80_ (BMIC_80_) of metronidazole for ATCC14018 was 16 μg/ml while that of clindamycin was 0.125 μg/ml. The minimum biofilm eradication concentration (MBEC) of metronidazole was > 256 μg/ml while that of clindamycin was > 2 μg/ml.

**Discussion:**

Our results revealed that *G. vaginalis* is more resistant to metronidazole than clindamycin and neither metronidazole nor clindamycin are able to effectively eradicate vaginal biofilms. Thus, the role of antibiotics and biofilms in BV requires further investigation.

## Introduction

Women of reproductive age from across the globe suffer from bacterial vaginosis (BV), a highly prevalent disease of the lower genital tract (Javed et al., 2019). Annually, $4.8 billion is spent on symptomatic BV treatment for 23–29% of women worldwide (Peebles et al., 2019). As a result of BV, women have an increased risk of adverse reproductive outcomes, gynecological complications and even HIV transmission (Hillier et al., 2021). Furthermore, there is a high recurrence rate for BV and this condition is difficult to cure (Bradshaw and Sobel, 2016).

BV is known to be caused by *Gardnerella vaginalis,* an anaerobic bacterium that is a resident in the normal vaginal flora of women. The vaginal flora is typically dominated by Lactobacilli species; however, BV can occur when organisms such as *G. vaginalis* grow excessively and become dominant (Zozaya-Hinchliffe et al., 2010). This bacterium was first discovered by Gardner and Dukes (1955) and named *G. vaginalis*. It is possible for this bacterium to be transmitted sexually between partners and to form biofilms that evade defense mechanisms of the host. The sexual transmission of the bacteria can alter the natural balance of the vaginal flora, thus resulting in the development of BV (Vodstrcil et al., 2017).

A biofilm consists of a structured community of microbes attached to a biological surface and encapsulated within a polymeric matrix consisting of carbohydrates, proteins and nucleic acids (Flemming et al., 2016; Jung et al., 2017). Biofilms promote the survival of *G. vaginalis* in the vagina. Compared to other anaerobes, *G. vaginalis* has a higher virulence, as characterized by higher levels of adhesion to epithelial cells and high levels of cytotoxicity (Alves et al., 2014). In contrast to planktonic bacteria, the biofilm of *G. vaginalis* is more resistant to two common agents that are present in healthy vaginal discharge: lactic acid and hydrogen peroxide. Chronic infections are often caused by microorganisms that can form biofilms (Wong et al., 2018). Biofilm-associated bacterial infections are characterized by elevated antibiotic resistance and extreme pathogenicity. In contrast, biofilms allow bacteria to evade host defense mechanisms and persist in the host for long periods of time (Warrier et al., 2021).

In general, *G. vaginalis* harbors several pathogenic factors, most notably sialidase and vaginolysin (VLY) (Hardy et al., 2017; Garcia et al., 2019). Sialidase breaks down the protective mucus layer of the vaginal epithelium by hydrolyzing sugar groups on the mucosa through sialic acid. This process may facilitate bacterial adhesion to the vaginal epithelium by interacting with other pathogenic organisms and is associated with the presence of biofilms (Schellenberg et al., 2016). VLY is a pore-forming toxic compound of the cholesterol-dependent cytolysin family that facilitates the lysis of target cells, such as vaginal epithelial cells. In the context of *G. vaginalis* infection, the *vly* gene encodes a pore toxin that affiliates with the human complement regulator CD59, a cytotoxin that assists in the initiation of *G. vaginalis* with host epithelial cells conglutination (Gelber et al., 2008).

Metronidazole and clindamycin are recommended as first-line therapies for *G. vaginalis* by the Centers for Disease Control and Prevention (CDC). However, relapse is a significant challenge for these current therapies; there is a > 50% recurrence rate due to the development of multispecies biofilms, in which *G. vaginalis* plays a dominant role (Machado et al., 2016).

In the present study, we aimed to investigate the prevlence of *sialidase* and *vaginolysin* encoding genes and determine the susceptibilities of planktonic *G. vaginalis* and biofilms to metronidazole and clindamycin. We also aimed to investigate the inhibition and eradication of *G. vaginalis* biofilm formation to provide an experimental basis for the clinical treatment of BV.

## Materials and methods

### Collection of patients specimens

The sampled population comprised 24 women who were randomly selected from the First Affiliated Hospital of Dalian Medical University between January 2019 and December 2019. The inclusion criteria were that the patient had not used systemic antibacterial or antifungal drugs within the last 30 days and had not engaged in sexual activity within 5 days of the examination. None of the patients had used topical vaginal products. Pregnant women and patients with other types of cervical-vaginal infections were excluded from the study. Vaginal specimens were collected during clinical examinations. For further experiments, sterile cotton swabs were saturated with vaginal secretions and sent to the clinical microbiology laboratory. Epithelial cells coated with bacteria were also visualized by Gram-stained smears on glass slides (clue cells). The study was performed in accordance with the relevant guidelines and regulations, and ethically approved by the ethical approval and consent of the First Affiliated Hospital of Dalian Medical University Ethics Committee (PJ-KS-KY-2022-294).

### Cultivation and identification of *Gardnerella vaginalis*

Incubation of the *G. vaginalis* samples was carried out anaerobically at 37°C for 48–72 h on Columbia agar (Oxoid) supplemented with sterile defibrinated sheep blood. Colonies were cultured in Brain Heart Infusion (BHI) broth supplemented with 2% (w/v) gelatin, 0.5% yeast extract, 0.1% starch and 1% glucose. *G. vaginalis* strain 14,018 was obtained from the American Type Culture Collection (ATCC). *G. vaginalis* samples were identified by their characteristic colony morphology and beta hemolysis on Columbia agar. Gram staining revealed gram-variable pleomorphic rods, and catalase testing was negative. Matrix-assisted laser desorption ionization time-of-flight mass spectrometry (MALDI-TOF MS) was carried out to identify *G. vaginalis*. The samples were then confirmed by 16S rDNA gene amplification and sequencing. The 16S rDNA gene was amplified by using specific primers: 27F (5′-AGTTTGATCCTGGCTCAG-3′) and 1492R (5′-GGTTACCTTGTTACGACTT-3′). To confirm the results, the 16 s rDNA sequences were compared with the Genbank library using the BLAST program.^1^ The *G. vaginalis* samples were stored at −80°C in De Man, Rogosa, and Sharpe (MRS) broth containing 30% glycerol.

### *Gardnerella vaginalis* clade-specific PCR assays

*Gardnerella vaginalis* clades were detected by the amplification of Gv1-fucosidase S and Gv1-fucosidase-AS primers, Gv2-hyp-S and Gv2-hyp-AS primers, Gv3-thi-S and Gv3-thi-AS primers and Gv4-cic-S and Gv4-cic-AS primers (Balashov et al., 2014). Bacterial genomic DNA was extracted from cultures with a Wizard Genomic DNA Purification Kit (Promega, Madison, United States) according to the manufacturer’s instructions. The reactions were performed in a final volume of 25 μl, containing 1.0 μl of each primer, 2.0 μl of DNA template and 12.5 μl of Premix Taq polymerase (Takara). The reaction mixture was subjected to 38 cycles of denaturation at 95°C for 30 s, primer annealing at 60°C for 30 s and extension at 72°C for 30 s. The last cycle included a 7-min extension step. PCR products were separated on 3.0% agarose gels stained with safe stain (Greenview Plus, Andy GoldTM, United States).

### Biofilm formation and biomass quantification

To quantify biofilm formation, the concentration of cultures after 48 h was adjusted to a final concentration of approximately 10^6^ CFU/ml. Biofilm biomass was quantified using the crystal violet (CV) staining method, as previously described by Peeters et al. (2008). In brief, the spent medium was removed after biofilm formation and 200 μl of phosphate buffered saline was added to each well to wash the preformed biofilms. The biofilms were then fixed with 100 μl of 99% (v/v) methanol (Sinopharm Chemical Reagent Company, Beijing, China) per well. The supernatants were removed after 15 min, and the microplates were then air-dried. Next, 100 μl of 0.5% (wt/v) CV (Kermal Chemical Reagent Company, Tianjin, China) was applied to the biofilm for 20 min. Subsequently, the excess CV was removed by washing the plates twice with 200 μl of phosphate buffer. Finally, the microplates were gently mixed after adding 150 μl of 33% (v/v) acetic acid (Kermal Chemical Reagent Company, Tianjin, China) per well to solubilize the CV. A 96-well microplate reader (Bio-Rad Laboratories, Hercules, CA, United States) was used to measure the optical density (OD) at 570 nm. All assays were repeated at least three times. Based on the cut-off OD value (ODc), defined as three standard deviations (SD) above the mean OD of the negative control, the samples were classified into four categories for biofilm formation (Stepanovic et al., 2000): OD ≤ ODc, no biofilm; OD ≤ 2 × ODc, weak biofilm; 2 × ODc < OD ≤ 4 × ODc, moderate biofilm; and OD > 4 × ODc, strong biofilm. The experiment was performed in triplicate.

### Confocal laser scanning microscopy analysis of biofilm

The formation of biofilms by *G. vaginalis* was investigated by CLSM. Biofilm staining was performed according to the manufacturer’s instructions in accordance with the LIVE/DEADTM Bac LightTM Bacterial Viability Kit (L13152, Invitrogen, United States) with some adjustments. In brief, the samples were cultured in 24-well plates (NEST Biotechnology Company, Wuxi, China) with a 15 mm × 15 mm circular cover glass (NEST Biotechnology Company, Wuxi, China) at the bottom of each well at 37°C under anaerobic conditions. After incubation, the coverslip was removed and washed three times with BHI; then, the coverslip was incubated for 25 min with 300 μl of fluorescent stain in the dark at 25°C. The staining solution was prepared with propidium iodide stain and SYTO®9 stain in 2.5 ml of ddH_2_O followed by CLSM (Leica, Germany) observation with an oil lens at 63 × magnification combined with 0.75 × zoom. Spectral Borealis lasers (green, 488 nm; red, 561 nm) were used for excitation. To obtain three-dimensional images, tomographic scans were performed at intervals of 1 mm in the Z-axis direction to obtain a series of images from each layer by using Leica SP8 software. Each sample was evaluated in five fields and the thickness of the biofilm was recorded.

### Scanning electron microscopy images of biofilm

The structure of the biofilm formed by *G. vaginalis* was visualized by scanning electron microscopy. We inoculated a single colony into the BHI medium for incubation under anaerobic conditions at 37°C. Then, we precipitated the bacteria by centrifugal collection; this was resuspended in PBS. After gently rinsing the precipitate three times, we used a pipette to aspirate the PBS and added 2.5% glutaraldehyde (the volume was more than 10 times that of the solid precipitate) for resuspension and mixed the bacterial precipitate in the fixative by pipetting at room temperature. After being fixed for 2 h, the suspension was progressively dehydrated using a graded series of ethanol solutions from 30 to 100%. After attaching to metallic stubs by carbon stickers, the samples were sputter-coated with gold for 30 s and the final SEM (Hitachi, Japan) images were acquired.

### Screening of the *vaginolysin* and *sld* genes by polymerase chain reaction

For the *vly* gene screening, we used the following primers: *vly*-585F 5’-GTACGATTCTGCAAGCGCACAAAGC-3′ and *vly*-1334R 5’-CCTTCCCAAGCGCGAGAACGC-3′. For the *sld* gene, we used the following primers: *sia1*F 5’-ATGGAACGTCGTTCAACGAAG-3′ and *sia1*R 5’-GATACGCGTTTTATGTCTCTTGC-3′. PCR reactions involved the DNA template (1.0 μl), 0.8 μl of each primer and 10 μl of PCR Taq DNA polymerase (Takara); this was made up to a final volume of 20 μl. Amplification of the *vly* and *sld* genes included initial denaturation at 94°C for 3 min followed by 28 cycles of denaturation at 94°C for 30 s, annealing at 52°C for 30 s, and extension at 72°C for 50 s, followed by a final extension at 72°C for 5 min. The amplicons in each reaction were analyzed on 2% agarose gel treated with safe stain (Greenview Plus, Andy GoldTM, United States) in 0.5X TAE after electrophoresis. The gels were visualized in a transilluminator with UV light (Tanon, China).

### Antimicrobial susceptibility testing of planktonic *Gardnerella vaginalis*

Vaginal samples were evaluated for susceptibility to metronidazole (Sigma-Aldrich, USA) and clindamycin (TCI, Japan) using the anaerobic micro-broth dilution method as described by Clinical and Laboratory Standards Institute (2012, 2018). The concentrations of antimicrobial clindamycin ranged from 0.0019 to 32 μg/ml, while those of metronidazole ranged from 0.031 to 32 μg/ml. Prior to testing, *G. vaginalis* was cultivated on Columbia agar with 5% sheep blood and incubated in anaerobic jars. The samples were suspended in BHI broth in a 0.5 McFarland suspension. The prepared modified BHI medium and antibacterial drug intermediate solution (10×) were added to each well of a 96-well cell culture plate at a ratio of 9:1 (90 μl:10 μl). Each plate included a row of wells without drugs as a control group. The prepared microdilution plate was packaged and immediately placed in a −80°C refrigerator for subsequent analysis. The lowest antibiotic concentration to no growth was read as the Minimum Inhibitory Concentration (MIC). The control isolate, *Bacteroides fragilis* ATCC 25285, was used to ensure quality control. *G. vaginalis* ATCC 14018 was also used as a supplemental control only when testing *G. vaginalis* samples. The microbiological susceptibility and resistant breakpoints for clindamycin (≤2 μg/ml and ≥ 8 μg/ml) and metronidazole (≤8 μg/ml and ≥ 32 μg/ml) were used to interpret the MIC results, as defined by Clinical and Laboratory Standards Institute (2012, 2018). The experiment was performed in triplicate.

### Antimicrobial susceptibility testing for *Gardnerella vaginalis* biofilms

Biofilms of *G. vaginalis* clinical samples and ATCC14018 were formed in 96-well plates. Metronidazole at concentrations ranging from 1 μg/ml to 256 μg/ml, clindamycin at concentrations ranging from 0.0019 μg/ml to 2 μg/ml were prepared in BHI medium and added to biofilm-containing microwells. After incubating anaerobically at 37°C for 48 h, the antimicrobial efficacy against biofilms was assessed by the XTT assay (Pettit et al., 2009; Yue et al., 2016). The inhibition percentage was calculated using the following formula: 100 (treated O.D. × 100/untreated O.D.) (Fernandes et al., 2020). The biofilm MIC_80_ (BMIC_80_) was determined as the lowest concentration that inhibited ≥ 80% of growth. The experiment was performed in biological triplicate.

### Minimum biofilm eradication concentration for *Gardnerella vaginalis* biofilms

For the biofilm eradication test, *G. vaginalis* was cultured in 96-well plates with BHI medium for 48 h. Biofilms were then washed three times with PBS. Metronidazole and clindamycin were then added. After incubating anaerobically at 37°C for 48 h, the broth recovery method was used to define the MBEC for *G. vaginalis* biofilms. To further verify the MBEC, the remaining biofilms were then swabbed with a cotton-tipped swab, cultured on a Columbia agar supplemented with sterile defibrinated sheep blood and incubated anaerobically for 3 days at 37°C. The MBEC was the minimum concentration resulting in no bacterial growth (Qu et al., 2010). The experiment was performed in biological triplicate.

### Biofilm inhibition and eradication of *Gardnerella vaginalis* toward metronidazole and clindamycin as observed by CLSM

Moderated biofilms including ATCC 14018 and weak biofilm samples were randomly selected and cultured in 24-well plates with a 15 mm × 15 mm circular cover glass at the bottom of each well at 37°C under anaerobic conditions. The medium was supplemented with metronidazole and clindamycin, respectively. These samples were then subjected to CLSM, as described earlier.

### Data analysis

Statistical analyzes were performed using GraphPad Prism v.8 software (GraphPad, La Jolla, CA, United States).

## Results

### *Gardnerella vaginalis* subgroups by clade-specific PCR

Clade 1 was the most frequently detected (75%) clade, followed by clade 4 (62.5%), clade 2 (20.8%) and clade 3 (4.2%). One of the samples (DL-23) did not belong to any clade that was detectable by clade-specific PCR but was identified as *G. vaginalis* by its characteristic microbiological profile and the nucleotide sequence of its *16S* rDNA coding gene.

### Total biomass quantification of *Gardnerella vaginalis*

**Figure 1:**
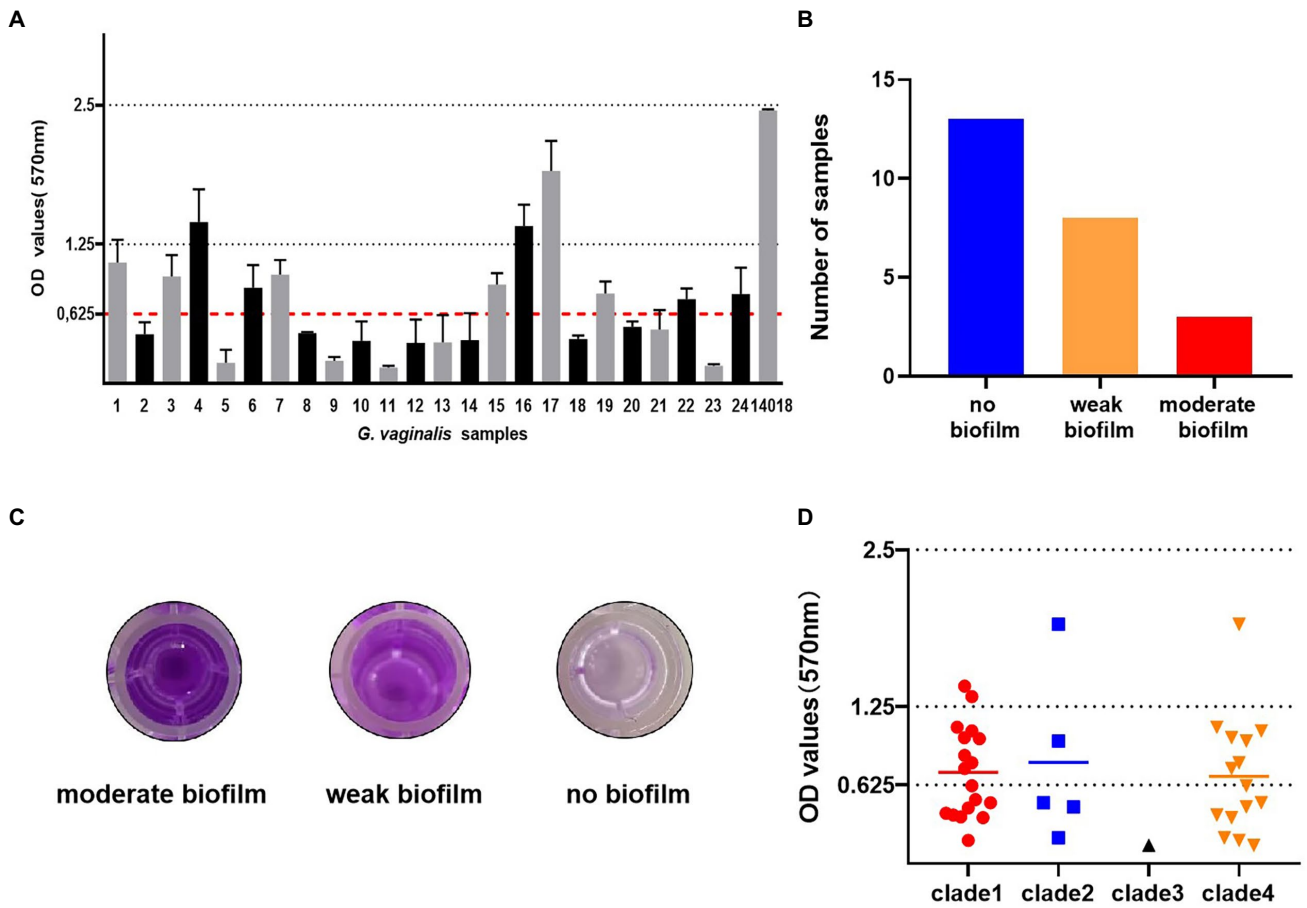
Biofilm formation of *G. vaginalis* clinical isolates and a laboratory reference strain. **(A)** The optical densities of *G. vaginalis* clinical isolates and ATCC14018. (The calculated cut-off optical density value was 0.625. no biofilm: < 0.625; weak biofilm: 0.625–1.25; moderate biofilm: 1.25–2.5). **(B)** The number of samples with moderate biofilm, weak biofilm and no biofilm. **(C)** The appearance of biofilms in 96-well microplates, as determined by crystal violet staining. **(D)** The relationship between biofilm formation and clades. Biofilms were stained with crystal violet and the amount of biofilm formation was measured at 570 nm.

All *G. vaginalis* samples were cultured in BHI medium in 96-well microplates for 24 and 48 h to evaluate biofilm formation *in vitro*. Biofilm formation ability was examined by crystal violet staining (Figure 1C). The calculated ODc value for this experiment was 0.625. Of the 24 samples of *G. vaginalis*, 11 samples and ATCC 14018 formed biofilms; the remainder did not (Figure 1A). Eight of the 11 samples (66.67%) produced a weak biofilm and three (33.33%) produced a moderate biofilm; none of the samples produced a strong biofilm (Figure 1B).

We observed biofilm formation in all clades (Figure 1D). The least frequently detected clade, clade 3, was only found in the non-biofilm group. The most ubiquitous clade, clade 1, was found in 69.2, 66.7, and 87.5% of the no biofilm group, weak biofilm group and moderate biofilm group, respectively. Clade 2 was the group most frequently observed in the moderate biofilm group.

### Morphological structure of biofilms as determined by CLSM and SEM

**Figure 2:**
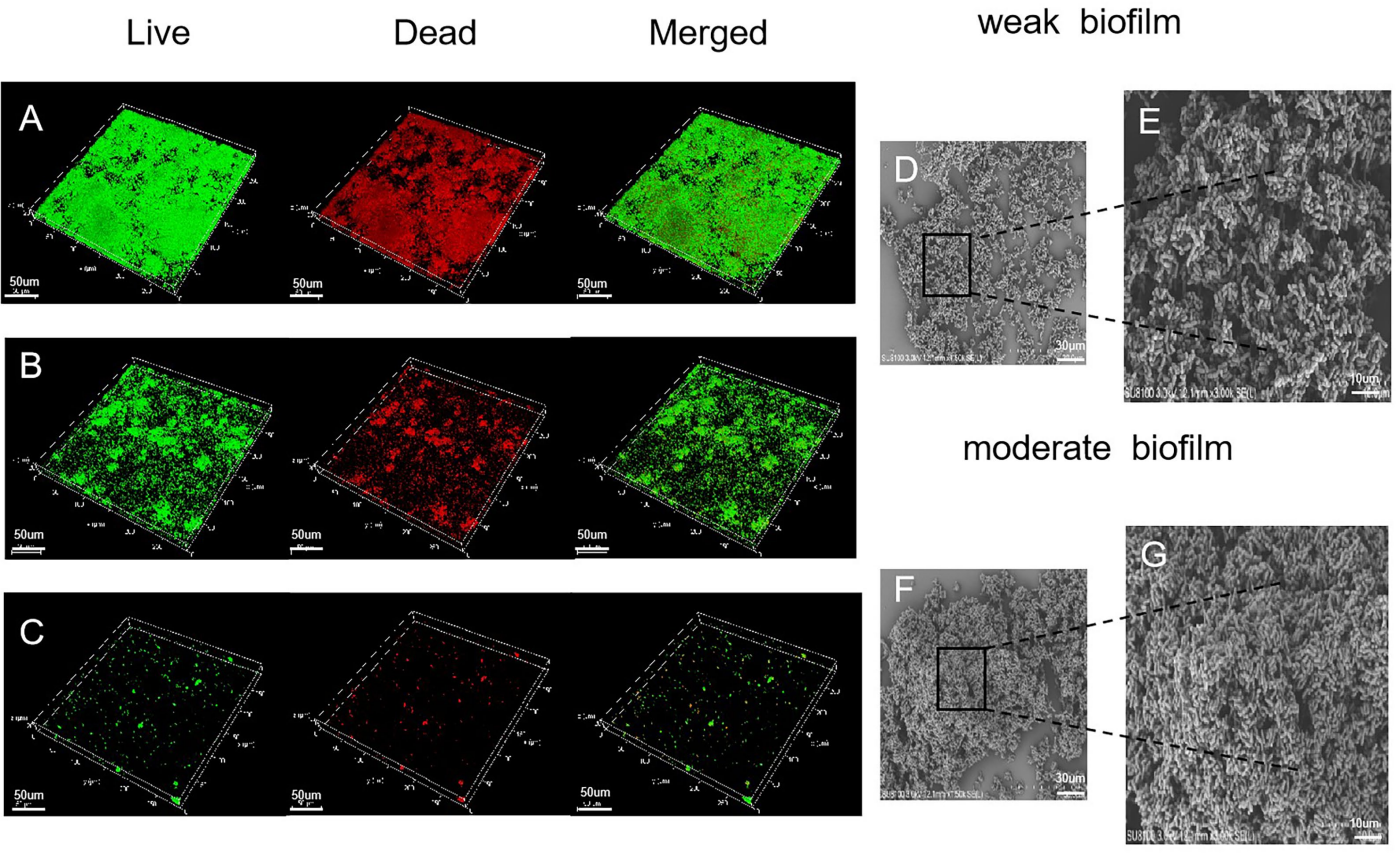
Microscopic structure of the biofilm, as determined by CLSM (×400 magnification). **(A)** Moderate biofilm detected by Live/Dead® viability staining; the green color indicates live cells and the red color indicates dead bacteria. **(B)** A strain that produced a weak biofilm. **(C)** A strain that produced no biofilm. **(D)** SEM images (×1,500 magnification) illustrating a weak biofilm. Scale bars represent 30 μm. **(E)** SEM images (×3,000 magnification) illustrating a weak biofilm. Scale bars represent 10 μm. **(F,G)** SEM images of moderate biofilm.

Biofilm structure was assessed by CLSM which allows the microstructure to be visualized in 5 μm horizontal optical slices. In the moderate biofilm samples, observations of the CLSM images of biofilm (Figure 2A) demonstrated that bacteria had aggregated into larger clumps with more live bacteria (green) than dead (red). In the weak biofilms, the CLSM images exhibited relatively weak ability to form biofilms; the biofilm thickness was not dense and showed black gaps (Figure 2B). In contrast, the dark background of the images (Figure 2C) indicated the non-fluorescent properties of the model materials while the scattered punctiform distribution indicated the lack of biofilm.

Electron microscopy was used to visualize the biofilms. The SEM images of *G. vaginalis* weak biofilm on platinum showed rod-shapes embedded in an extracellular polymeric substance (Figure 2D). The *G. vaginalis* formed complex clusters (Figure 2E). The moderate biofilms showed dense bacterial clumps with a complex and stereospecific film structure, embedded in extracellular polymeric substances (Figures 2F,G).

### Detection of the *vaginolysin* and *sialidase a* genes

**Figure 3:**
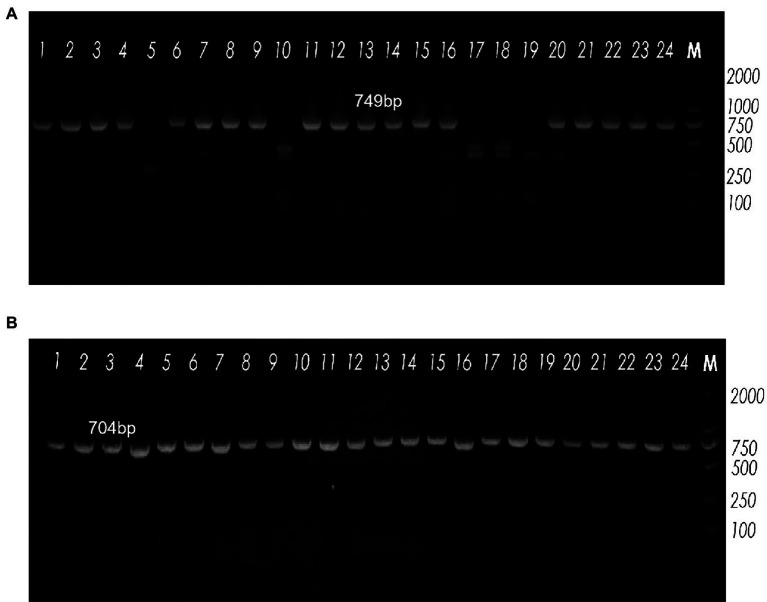
Agarose gel electrophoresis showing band patterns. **(A)** The expression levels of the *vly* gene, as demonstrated by agarose gel electrophoresis. Lane M: DNA marker (DL2000); Lanes 5, 10, 17, 18 and 19: DNA samples negative for the *vly* gene; Lanes 1−4, 6−9, 11−16, 20–24: DNA samples positive for the *vly* gene. **(B)** The expression levels of the *sialidase A* gene, as detected by agarose gel electrophoresis. Lane M: DNA marker (DL2000); all lanes were positive for the *sialidase A* gene.

Vaginolysin (VLY), produced by *G. vaginalis*, is a cholesterol-dependent cytolysin (CDC) that may play a role as a virulence factor (Pleckaityte, 2020). The level of VLY secretion, which varies among *G. vaginalis* samples, may correlate with the severity of bacterial vaginosis. To amplify the toxin vaginolysin coding gene (*vly*), we designed two primers: *vly*-585F and *vly*-1334R (Figure 3A). We found that 19 samples (79.2%) tested positive by *vly* PCR. The *sialidase* virulence gene in *G. vaginalis* encodes an enzyme associated with host bacterial invasion, which enzymatically eliminates terminal sialic acid residues from different glycoconjugates, thus improving the bacteria’s ability to evade the host’s immune system and manipulate cellular interactions. ATCC 14018 was used as a positive control for the *sialidase A* gene; the positive band was approximately 704 bp in size. Analysis showed that all samples carried the *sialidase A* gene (100%, Figure 3B).

### Antibacterial activities of clindamycin and metronidazole against planktonic *Gardnerella vaginalis in vitro*

The 24 *G. vaginalis* samples and ATCC 14018 were evaluated for their susceptibility to clindamycin and metronidazole. The concentration ranges of metronidazole and clindamycin were 0.031–32 μg/ml and 0.0019–32 μg/ml, respectively. MIC breakpoints were defined by CLSI criteria for metronidazole (≤8 μg/ml and ≥ 32 μg/ml) and clindamycin (≤2 μg/ml and ≥ 8 μg/ml). As summarized in Table 1, 21 (87.5%) of the samples showed a high resistance to metronidazole; only DL-14 and ATCC 14018, with a MIC threshold of 8, were sensitive to metronidazole. Moreover, DL-1 and DL-10 were intermediately resistant to metronidazole. For clindamycin, as shown in Table 1, 16 samples (67%) presented high sensitivity toward clindamycin, of which the antibacterial ability was significantly higher than metronidazole. These data showed that *G. vaginalis* samples collected from Northeast China are more resistant to the clinical first-line drug metronidazole than clindamycin in this region.

**Table 1:** Summary of antimicrobial sensitivity to *G. vaginalis* clinical samples grown as planktonic cells.

Sample no.	Biofilm	Clade by qPCR	Metronidazole (μg/ml)	Clindamycin (μg/ml)
			MIC	MIC
DL-1	Weak	1,4	16	0.0625
DL-2	Negative	1	≥32	0.0625
DL-3	Weak	1,4	≥32	0.031
DL-4	Moderate	1	≥32	0.031
DL-5	Negative	1,4	≥32	0.015
DL-6	Weak	1	≥32	0.0625
DL-7	Weak	2,4	≥32	0.031
DL-8	Negative	2,4	≥32	0.0625
DL-9	Negative	2,4	≥32	≥32
DL-10	Negative	1,4	16	0.0625
DL-11	Negative	3,4	≥32	≥32
DL-12	Negative	1,4	≥32	0.0625
DL-13	negative	1	≥32	≥32
DL-14	Negative	1,4	8	0.015
DL-15	Weak	1	≥32	≥32
DL-16	Moderate	1	≥32	≥32
DL-17	Moderate	2,4	≥32	0.031
DL-18	Negative	1	≥32	0.5
DL-19	Weak	1,4	≥32	0.015
DL-20	Negative	1	≥32	0.015
DL-21	Negative	1,2,4	≥32	≥32
DL-22	Weak	1,4	≥32	≥32
DL-23	Negative	ND	≥32	≥32
DL-24	Weak	1,4	≥32	0.0625
ATCC14018	Moderate	1	8	0.0625

### Biofilm inhibition of *Gardnerella vaginalis* toward metronidazole and clindamycin

**Figure 4:**
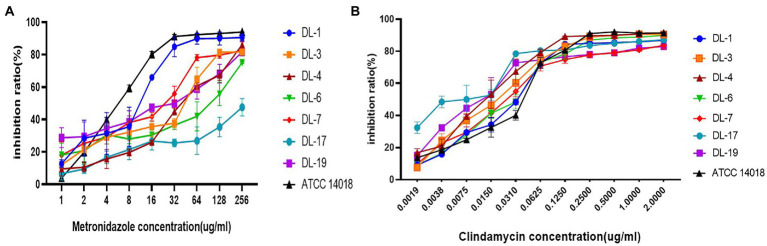
Biofilm inhibition rates of *G. vaginalis* in response to metronidazole and clindamycin. **(A)** Inhibition rates of metronidazole on the formation of biofilm by various samples. **(B)** Inhibition rates of clindamycin.

Seven *G. vaginalis* samples (weak biofilm: DL-1, 3, 5, 6 and 19; moderate biofilm: DL-4 and 17) and ATCC 14018 were used to investigate the changes of inhibition rates in the presence of metronidazole and clindamycin. With an increase of drug concentration, the inhibition rate of biofilm gradually increased. The inhibition rate of DL-1 remained above 80% when the metronidazole concentration was 32 μg/ml. The inhibition rate of DL-17 did not exceed 50% despite the concentration of metronidazole increasing to 256 μg/ml (Figure 4A). All the inhibition rates of *G. vaginalis* biofilm exceeded 80% when the clindamycin concentration reached 2 μg/ml (Figure 4B).

Compared with the planktonic MIC, the BMIC_80_ was at least two-fold higher (Table 2). The BMIC_80_ of metronidazole on ATCC14018 was 16 μg/ml; for clindamycin, the BMIC_80_ was 0.125 μg/ml.

**Table 2:** Antimicrobial susceptibility of *G. vaginalis* clinical samples grown as biofilms.

Sample no.	Biofilm	Metronidazole (μg/ml)		Clindamycin (μg/ml)	
		BMIC_80_	MBEC	BMIC_80_	MBEC
DL-1	Weak	32	>256	0.125	1
DL-3	Weak	256	>256	0.0625	1
DL-4	Moderate	256	>256	0.0625	>2
DL-6	Weak	>256	>256	0.125	1
DL-7	Weak	128	>256	0.0625	2
DL-17	Moderate	>256	>256	0.0625	2
DL-19	Weak	>256	>256	0.031	>2
ATCC14018	Moderate	16	>256	0.125	>2

### Minimum biofilm eradication concentration for *Gardnerella vaginalis* biofilms

Seven *G. vaginalis* samples (weak biofilm: DL-1, 3, 5, 6 and 19; moderate biofilm: DL-4 and 17) and ATCC 14018 were used to investigate the changes of biofilm eradication in the presence of metronidazole and clindamycin. With regards to metronidazole and clindamycin, broth recovery-based biofilm MBECs were much higher than MICs (Table 2). The MBEC of metronidazole on ATCC14018 was > 256 μg/ml while that for clindamycin was > 2 μg/ml.

### Biofilm inhibition and eradication by CLSM

**Figure 5:**
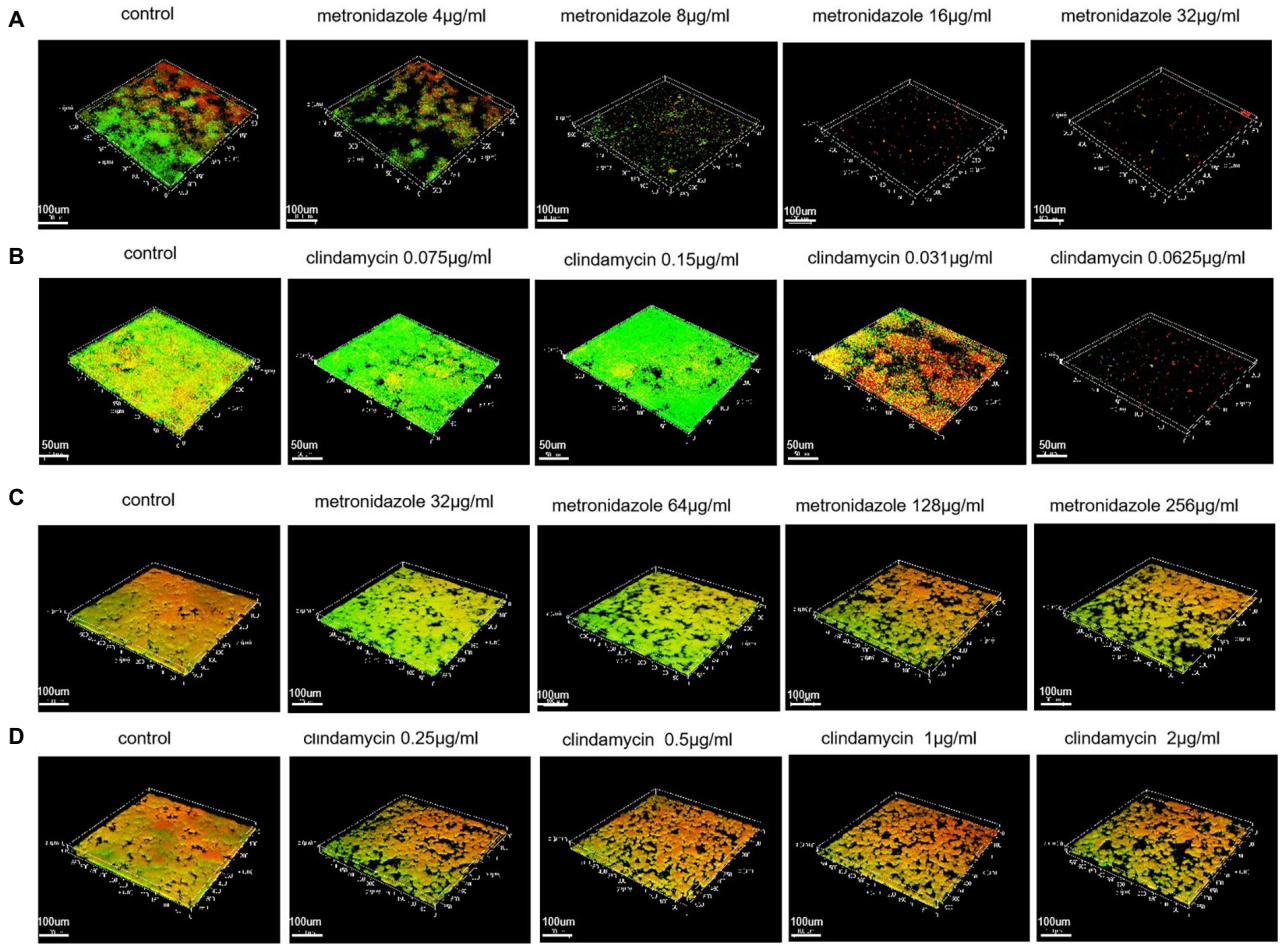
Biofilms inhibition and eradication detected with the Live/Dead® Viability Kit and CLSM. **(A)** Biofilms inhibition of ATCC 14018 at different metronidazole concentrations (control; 4 μg/ml; 8 μg/ml; 16 μg/ml and 32 μg/ml). **(B)** Biofilms inhibition of ATCC 14018 at different concentrations of clindamycin (control; 0.0075 μg/ml; 0.015 μg/ml; 0.031 μg/ml and 0.0625 μg/ml). **(C)** Biofilm eradication of ATCC 14018 under different concentrations of metronidazole (control; 32 μg/ml; 64 μg/ml; 128 μg/ml and 256 μg/ml). **(D)** Biofilm eradication of ATCC 14018 at different clindamycin concentrations (control; 0.25 μg/ml; 0.5 μg/ml; 1 μg/ml and 2 μg/ml).

Based on the results of biofilm inhibition rates we have detected, CLSM was used to visualize the growth of bacterial colonies (both with intact membranes and damaged membranes) under the effect of different drug concentrations. With an increase of drug concentration, the biofilm formation ability gradually weakened until no biofilm was formed (Figures 5A,B).

With regards to the eradication test, CLSM investigation showed there was a general decrease in bacterial aggregation with an increase in drug concentration. However, until the metronidazole concentration reached 256 μg/ml and the concentration of clindamycin reached 2 μg/ml, the biofilm of ATCC14018 was not effectively removed (Figures 5C,D).

## Discussion

In the Human Microbiome Project, the vaginal microbiome has been demonstrated to be unique in terms of its microbial diversity (Kroon et al., 2018). BV can occur when *Lactobacillus* spp. are absent from the vaginal environment (Muzny et al., 2020). It is well known that highly structured polymicrobial biofilms can form on the vaginal epithelium in patients with BV and *G. vaginalis* is often the predominant species (Machado and Cerca, 2015).

As communities of microorganisms attached to surfaces, biofilms play an important role in the persistence of bacterial infections (Del Pozo, 2018). A biofilm contains bacteria that are much more resistant to antibiotics than planktonic bacteria. The study of bacterial biofilms can provide new concepts for clinical treatment. The biofilm formed by these bacteria is equivalent to a physical barrier that can effectively resist the antibiotic attack. Consequently, there is some correlation between the ability of biofilm formation and bacterial drug resistance. Clarification of the state of biofilm formation in the different samples that were isolated and cultured from vaginal-swab specimens is of great guidance for clinical drug application. Our results suggested that 11 (45.83%) of the 24 *G. vaginalis* samples and ATCC14018 could form biofilms, thus indicating that *G. vaginalis* has a certain biofilm-forming ability. The complex three-dimensional structure of the biofilm was visualized by CLSM. Some samples that were categorized as moderate biofilm could generate more compact and thicker biofilm structures. A dense cluster of rods embedded in an extracellular matrix was also observed in *G. vaginalis* biofilms by SEM.

The analysis of *G. vaginalis* clade distribution in 24 samples by multiplex clade-specific PCR revealed a dominance of clade 1 (75%), followed by the less frequent clade 4 (62.5%), clade 2 (20.8%) and clade 3 (4.2%). It is possible that individuals were infected with multiple strains of *G. vaginalis* and due to technical difficulties we were unable to identify single unique isolates in these patients. Clade 1 and clade 4 were the most common; this is consistent with the previous findings of Janulaitiene et al. (2017). Some studies indicated that clades 1, 2 and 3 were significantly associated with current sexual practices (Plummer et al., 2020). Previous findings suggested that the multiple clade structure was caused by unprotected sex with new partners (Balashov et al., 2014). However, it should be noted that the correlation between *G. vaginalis* clades and biofilm formation remains unclear. Moreover, the biofilm of *G. vaginalis* can be exchanged between sexual partners. Heterosexual couples have been shown to share identical *G. vaginalis* strains and demonstrated high concordance for *G. vaginalis* biofilm (Swidsinski et al., 2010)_._ The isolation of *G. vaginalis* samples of an unknown subtype (DL-23) underscores the high complexity of the *Gardnerella* genus; this requires further investigation. Culture-based approaches to study *G. vaginalis* represent a notable technical challenge.

In this study, we analyzed *G. vaginalis* with respect to the genes that secrete active sialidase and vaginolysin. *Sialidase,* as a virulence gene that removes terminal sialic acid residues from different glycoconjugates, is considered to be relevant to the condition of the biofilm formation. The sialidase produced by *G. vaginalis* promotes the depletion of mucus and the degradation of secretory immunoglobulin A in both *in vivo* and *in vitro* studies (Lewis et al., 2013). Santiago (Lopes et al., 2011) previously showed that almost all strains with *sialidase* gene positivity were also positive for sialidase activity. In this study, we found that 100% of *G. vaginalis* samples collected from Northeast China were positive for the presence of the *sialidase A* gene; this was significantly higher than those reported by Shipitsyna et al. (2019). These results suggested that these samples might all possess the ability to degrade mucus barriers. The reason for this may be the diversity of the *G. vaginalis* genotype and phenotype in different regions and populations. VLY is a secreted protein toxin that functions as a hemolysin specific for erythrocytes, neutrophils and endothelial cells, which interacts with CD59 involved in the pathogenesis of BV and consequent outcomes. The level of VLY secretion, which varies among *G. vaginalis*, may correlate with the severity of bacterial vaginosis. The positive rate of *vly* gene detection in the 24 *G. vaginalis* samples was 79.2%; this was higher than in other studies (Knupp de Souza et al., 2016; Mohammadzadeh et al., 2019).

Metronidazole and clindamycin are conventional medications that are clinically used in the treatment of bacterial vaginitis. The results of drug sensitivity to *G. vaginalis* samples and ATCC14018 showed that the resistance rates to metronidazole reached 87.5% (MIC_50_ = 32 μg/ml; MIC_90_ = 32 μg/ml); for clindamycin, resistance rates reached 33.3% (MIC_50_ = 0.0625 μg/ml; MIC_90_ = 32 μg/ml). Differences appear to exist between the mechanisms of action *in vivo* and *in vitro*, although these results are significant in that they can reveal the adverse habits of antibiotic use and the extremely high levels of bacterial resistance in this region. Thus, conventional antibiotic therapy has limited function in the clearance of *G. vaginalis*.

The formation of biofilm is the main cause of BV recurrence. In addition, biofilm formation is associated with an increase in antimicrobial resistance and the recurrence of disease; it also creates the possibility for sexual transmission (Swidsinski et al., 2014). We measured the BMIC_80_ and MBEC of metronidazole and clindamycin against *G. vaginalis* by XTT and broth recovery-based methods. The efficacy of metronidazole and clindamycin against biofilms was confirmed by CLSM. When the drug concentration reached the highest drug concentration in the experiment (256 μg/ml of metronidazole and 2 μg/ml of clindamycin), the biofilm inhibition rates of metronidazole and clindamycin on *G. vaginalis* samples were lower than those on ATCC14018. The MIC and BMIC_80_ of metronidazole and clindamycin against ATCC14018, moderate biofilm and weak biofilm were compared; the results showed that the BMIC_80_ was two-fold higher than the planktonic MIC at least. When the drug concentration reached the MIC for bacterial growth, there was still a small amount of biofilm in the bacteria, thus indicating that the clinical drug concentration only inhibited the growth of bacteria; the bacterial biofilm was not affected. The same strain had stronger drug resistance than planktonic bacteria after biofilm formation. With regards to MBEC, which defines the concentration to definitively eradicate biofilm cells (100% kill), is necessary for the successful treatment of biofilm-related infections (Qu et al., 2010). However, the highest experimental concentrations of metronidazole and clindamycin (256 μg/ml for metronidazole and 2 μg/ml for clindamycin) could not effectively eradicate the formed biofilm. A small fraction of bacteria in biofilms still survived antibiotic killing. Similar to many other biofilm-related infections, standard antibiotics such as metronidazole and clindamycin are unable to eradicate vaginal biofilms effectively (Fux et al., 2005). In this study, both the XTT and broth recovery-based assays were associated with certain limitations with regards to the biofilm inhibition and eradication experiments. The biofilms cultured *in vitro* grew in a suitable environment and adhered to the solid surface without external interference. However, there are many other microorganisms involved and limiting factors in the dynamic process of *G. vaginalis* from adhesion to vaginal epithelium to biofilm maturation. Moreover, viable bacteria cannot be measured, and the drug action time is relatively short. Thus far, no drugs are in clinical use that specifically targets bacterial biofilms. This is probably because until recently the molecular details of biofilm formation were still poorly understood. The role of antibiotics and biofilms needs to be further studied.

## Conclusion

Our results reveal that the positive detection rates of the *sialidase A* gene and *vly* gene for *G. vaginalis* samples in Northeast China were higher than those reported in other studies. *G. vaginalis* is more resistant to metronidazole than clindamycin and neither metronidazole nor clindamycin could effectively eradicate vaginal biofilms. The role of antibiotics and biofilms needs to be investigated in more detail.

## Data availability statement

The data presented in the study are deposited in the GenBank repository, accession number OP984336-OP984359.

## Author contributions

XM and NW: conceptualization. NW, JL, and SY: methodology. XW: software and formal analysis. XM, NW, and SY: validation. NW: investigation, project administration, funding acquisition, and writing—review and editing. HY: resources. JL: data curation and writing—original draft preparation. HY: visualization. XM: supervision. All authors have read and agreed to the published version of the manuscript.

## Funding

This work was supported by grants from the Liaoning Province Science and Technology Project (grant no. 20180550729).

## Conflict of interest

The authors declare that the research was conducted in the absence of any commercial or financial relationships that could be construed as a potential conflict of interest.

## Publisher’s note

All claims expressed in this article are solely those of the authors and do not necessarily represent those of their affiliated organizations, or those of the publisher, the editors and the reviewers. Any product that may be evaluated in this article, or claim that may be made by its manufacturer, is not guaranteed or endorsed by the publisher.

## Footnotes

^1^ https://blast.ncbi.nlm.nih.gov
